# Survival in the Third Decade of Uncemented Total Hip Arthroplasty With Small-Diameter Metal-on-Metal Versus Ceramic-on-Conventional Polyethylene Bearings

**DOI:** 10.1016/j.artd.2026.102078

**Published:** 2026-07-08

**Authors:** Sandro Beros, Philemon Grimm, Michel Schläppi, Robin Pourzal, Joshua J. Jacobs, Peter Wahl

**Affiliations:** a Division of Orthopaedics and Traumatology, Cantonal Hospital Winterthur, Winterthur, Switzerland; b Division of Orthopaedics and Traumatology, Hospital Triemli, Zurich, Switzerland; c Department of Orthopedic Surgery, Rush University Medical Center, Chicago, IL USA; d Department of Biomedical Engineering, University of Basel, Allschwil, Switzerland; e ARTORG Center for Biomedical Engineering Research, Faculty of Medicine, University of Bern, Bern, Switzerland

**Keywords:** Total hip arthroplasty, Survival, Long-term, Revision rate, Metal-on-metal, Ceramic-on-conventional polyethylene

## Abstract

**Background:**

This study aimed to assess the long-term revision and mortality rates after uncemented total hip arthroplasty with small-diameter metal-on-metal (SD-MoM) and ceramic-on-conventional polyethylene (CoPE) bearings over 20-24 years of follow-up.

**Methods:**

This retrospective, single-center study included 622 total hip arthroplasty (1998-2001) with 3 stem (BEO, CLS Spotorno, Alloclassic) and 2 bearing types (SD-MoM: n = 458; CoPE: n = 164). Kaplan-Meier survival analysis assessed implant revision and patient mortality. Revision was defined as any surgical intervention involving the exchange, removal, or addition of implant components.

**Results:**

Revision rates did not differ significantly between SD-MoM and CoPE bearings (*P* = .77). SD-MoM bearings showed a steady increase in revisions, while CoPE bearings experienced a marked rise after 10 years, surpassing SD-MoM rates. Mortality was significantly lower in the SD-MoM group (19.9% vs 48.9% for CoPE at 20 years, *P* < .0001). No significant differences in revision rates were observed between the Alloclassic, CLS Spotorno stem and/or BEO stems.

**Conclusions:**

While SD-MoM bearings showed favorable long-term revision rates compared to CoPE, recent data suggest conflicting results. No increased mortality could be observed for SD-MoM, the even lower mortality in this group was likely due to the younger age of these patients. While SD-MoM seem to compare favorably to large diameter MoM regarding long-term outcomes, need for follow-up, including monitoring of cobalt and chromium blood levels, remains.

## Introduction

Total hip arthroplasty (THA) is very successful at relieving symptoms and impairment from a variety of end-stage hip joint diseases [[1], [2], [3], [4]]. It was even shown that cognitive functions improve after THA in patients suffering from hip osteoarthritis [5]. Short- and mid-term revision rates should be low. Nowadays, regulations in the UK even require using implants for routine care with revision rates below 5% at 10 years [6]. The risk for revision, however, increases over time, reaching 20 to 25% at 20 years and 30-35% at 30 years [[7], [8], [9]]. National arthroplasty registries mostly provide such data. Long-term observational studies after THA remain rare [7,8,[10], [11], [12], [13]]. While many factors influence the long-term outcomes after THA, uncemented stems are particularly affected by periprosthetic fractures, occurring 7-10 times more frequently than with cemented stems, both during the early phase as well as in the long term [14]. Uncemented implants, relying on integration by vital tissue, do not experience loosening due to material failure as it can be the case with bone cement [15].

At our institution, the surgical approach and the implants used for THA remained largely consistent throughout the 1990s and early 2000s. The choice of the bearing, either small diameter (SD) previously defined by De Steiger et al. as ≤ 32 mm, metal-on-metal (MoM) or alumina ceramics-on-conventional polyethylene (CoPE), was left to the surgeon, offering further opportunities for comparison [16]. In our cohort, solely 28-mm heads were used for all bearings. Thorough case coding and routine follow-up examinations provided an exceptional opportunity for long-term analysis. The recording of long-term revisions was also eased by the introduction of the Swiss National Joint Replacement Registry in 2012 [17]. As a rather large cohort of patients with SD-MoM THA was available, the analysis could also be extended to patient survival, as there may be systemic toxicity issues with MoM bearings, not necessarily identifiable through revision rates [[18], [19], [20], [21], [22]]. The aim of the present study was to answer the following questions:

- What is the long-term revision rate of uncemented THA in this cohort?
- How are long-term revision rates impacted by the use of different stems (in this cohort, the Alloclassic, the CLS Spotorno, and its subvariant, the BEO)?
- Is there a difference in the long-term revision rate depending on the bearing used (CoPE vs SD-MoM)?
- Does mortality over time differ between THA patients with SD-MoM compared to CoPE bearings?

## Material and methods

**Figure 1:**
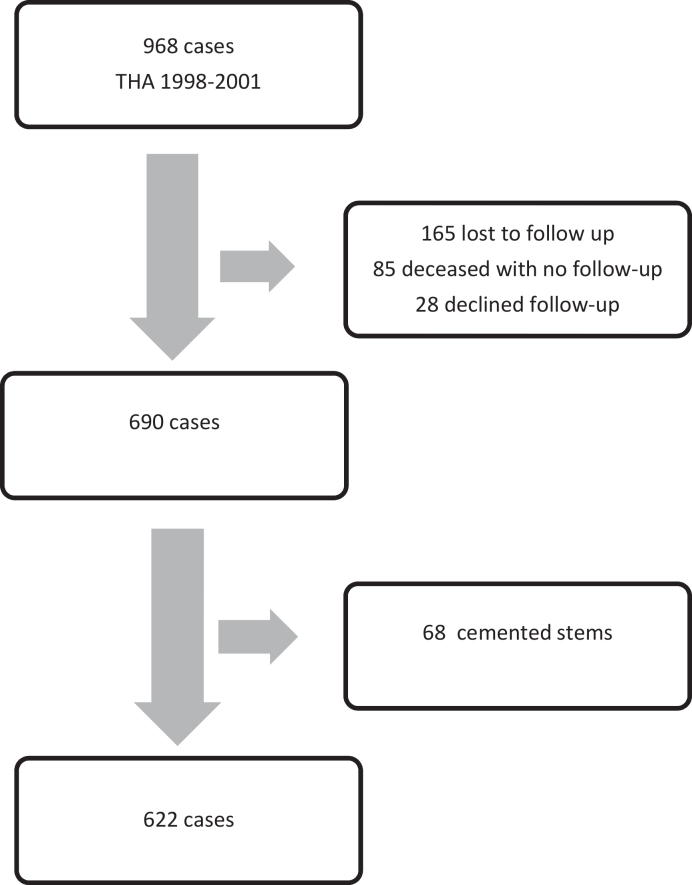
Patient selection. Patient selection flowchart. Excluded were patients without any follow-up data available and who were not reachable (n = 165), those who were deceased without any follow-up (n = 85), and those who declined routine follow-up (n = 28). Of the 690 cases remaining (627 patients), further 68 had to be excluded because of use of other stems than CLS Spotorno, BEO, or Alloclassic. Finally, 622 cases (522 patients) could be included in the analysis.

This was a retrospective, single-center study. All patients who underwent THA at our institution, between 1998 and 2001 included were selected to constitute the study cohort. Patient identification was performed through a code search of the electronic medical record system used at that time (HISS, Vitodata, Seuzach, Switzerland). Datasets from a total of 968 cases were identified. Of these, 622 cases, involving 522 patients, were eligible, after the exclusion of cases with cemented stems and of those with no follow-up data available (Fig. 1). Data extraction was performed from the medical files available in HISS, as well as in the successor system Phoenix (CGM, Coblenz, Germany). Date of implantation, basic demographic data, implant type, date of last follow-up, respectively, of revision or death, were registered in a specific in-house database, with a patient-specific code to ensure data anonymization. Revision was defined as usual as any operation with addition, removal, or exchange of a component of the THA. Revisions and patient death registered in-house as well as in the Swiss national arthroplasty registry Swiss National Joint Replacement Registry were considered [17]. Internal fixation due to a periprosthetic fracture led to censoring of the case, as these cases underwent only fracture fixation without any component exchange.

**Figure 2:**
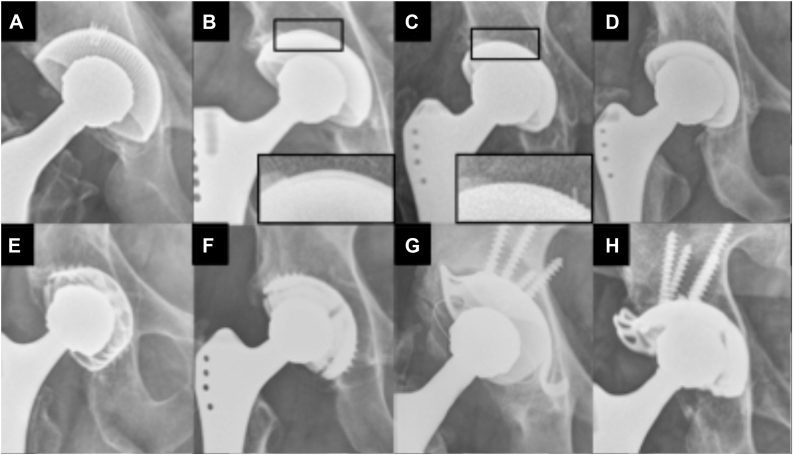
Overview of different Metasul metal-polyethylene-sandwich acetabular components. The images above are illustrative examples of Metasul bearings and do not necessarily represent cases from the current cohort. In our cohort, only Fitek/Fitmore pressfit cups and Mueller low profile cemented cup were used. (a) A Morsher monoblock cup. (b) A Fitek cup with fins. In the magnified view, the radiolucent line between the cup and the Sulmesh is clearly visible, distinguishing it from a Fitmore cup. (c) A Fitmore cup with fins and a magnified view. (d) A Allofit without screw holes. (e) An Alloclassic threaded cup. (f) A CLS Spotorno expansion shell. (g) A Ganz acetabular roof reinforcement ring with hook paired with a cemented Mueller low profile cup. (h) An original M.E. Mueller acetabular roof reinforcement ring with the corresponding Metasul inlay. The angular edge profile of the acetabular insert displays the polyethylene-metal-sandwich socket of the Metasul design.

The standard approach was transgluteal, performed in lateral position [23]. Uncemented implants were used routinely, with the Fitek/Fitmore or the cemented Mueller low profile cups and both the Alloclassic as well as the CLS Spotorno stem, including its BEO variant (all from Zimmer Biomet, Zug, Switzerland, previously Sulzer, respectively, Centerpulse, Winterthur, Switzerland). Cemented Mueller Straight stems were used only in case of strong indication for cement fixation of the stem, at the discretion of the surgeon. SD-MoM bearings had a polyethylene-metal-sandwich socket Metasul (Zimmer Biomet) (examples illustrated in Fig. 2) and CoPE bearings were Sulox heads articulating with Sulene sockets (Zimmer Biomet). Solely 28-mm heads were used for all bearings. SD-MoM bearings were favored for younger and more active patients, but the final decision was left to the surgeon.

Descriptive statistics were used to summarize patient demographics and implant types. Continuous data are presented as mean ± standard deviation (SD), or median (range), depending on data distribution. Categorical data are expressed as counts and percentages. A Kaplan-Meier survival analysis was conducted to compare the revision and mortality rates over time based on several categorical variables such as stem and bearing type. Ninety-five percent confidence intervals (95% CIs) were calculated for all survival estimates. Survival curves were compared using a log-rank test. To adjust for potential confounding by baseline characteristics, a multivariable Cox proportional hazards model were applied for both revision and mortality rates. Age and sex were included as covariates in both models. Adjusted hazard ratios (HRs) with 95% CI were calculated for bearing type and other covariates. The significance level was set at *P* = .05. All statistical analyses were performed using R version 4.2.0 (R Foundation for Statistical Computing, Vienna, Austria) with the packages survival (version 2.11.4) and survminer (version 0.4.9).

Routine follow-up monitoring of cobalt and chromium blood levels was performed routinely only from 2015 onward. No follow-up examination, no sampling, and no intervention were performed for study reasons. Only anonymized data were analyzed. The study protocol was accepted by the regional ethical committee (BASEC-Nr. 2023-01543).

## Results

The patient selection flowchart is illustrated in Figure 1. This cohort of uncemented THA included more males than females (352:270, 56.6%:43.4%). Alloclassic stems were used in 227 cases (36.5%), BEO stems in 159 cases (25.6%), and CLS Spotorno stems in 236 cases (37.9%). Fitek/Fitmore cups were used in 601 cases (96.6%), while 21 cases (3.4%) had an acetabular reinforcement ring with a cemented Mueller low profile cup. SD-MoM was used in 458 cases (73.6%) and CoPE in 164 cases (26.4%). The detailed numerical values as well as the patient characteristics can be found in Table 1. The SD-MoM cohort was significantly younger than CoPE patients (median 61.9 vs 74.2 years, *P* < .001) and consisted of significantly more males (60.7% vs 45.1, *P* < .001).

**Table 1:** Demographic and implant characteristics.

Parameter	Overall	BEO	CLS Spotorno	Alloclassic	CLS Spotorno and BEO	SD-MoM	CoPE
Cases, n (%)	622	159 (25.6)	236 (37.9)	227 (36.5)	395 (63.5)	458 (73.6)	164 (26.4)
Age, y median (range)	64.1 (26.7 – 84.9)	63.5 (45.5-84.9)	63.3 (26.7-73.0)	67.7 (31.9-83.6)	63.4 (26.7-84.9)	61.9 (26.7-80.4)	74.2 (41.9 -84.9)
Sex							
Male	352 (56.6)	136 (85.5)	120 (50.8)	96 (42.3)	256 (64.8)	278 (60.7)	74 (45.1)
Female	270 (43.4)	23 (14.5)	116 (49.2)	131 (57.7)	139 (35.2)	180 (39.3)	90 (54.9)
Side (%)							
Right	306 (49.2)	78 (49.1)	120 (50.8)	108 (47.6)	198 (50.1)	232 (50.7)	74 (45.1)
Left	316 (50.8)	81 (50.9)	116 (49.2)	119 (52.4)	197 (49.9)	226 (49.3)	90 (54.9)
Cup type							
Fitmore	601 (96.6)	159 (100)	232 (98.3)	210 (92.5)	391 (99.0)	446 (97.4)	155 (94.5)
Reinforcement ring with cemented Mueller low profile	21 (3.4)	0	4 (1.7)	17 (7.5)	4 (1.0)	12 (2.6)	9 (5.5)
Bearing							
SD-MoM	458 (73.6)	127 (79.9)	188 (79.7)	143 (63.0)	315 (79.7)	458 (100)	0
CoPE	164 (26.4)	32 (20.1)	48 (20.3)	84 (37.0)	80 (20.3)	0	164 (100)

Summary of the demographic and clinical characteristics of the 622 cases included in the study, categorized by stem and bearing type.

**Figure 3:**
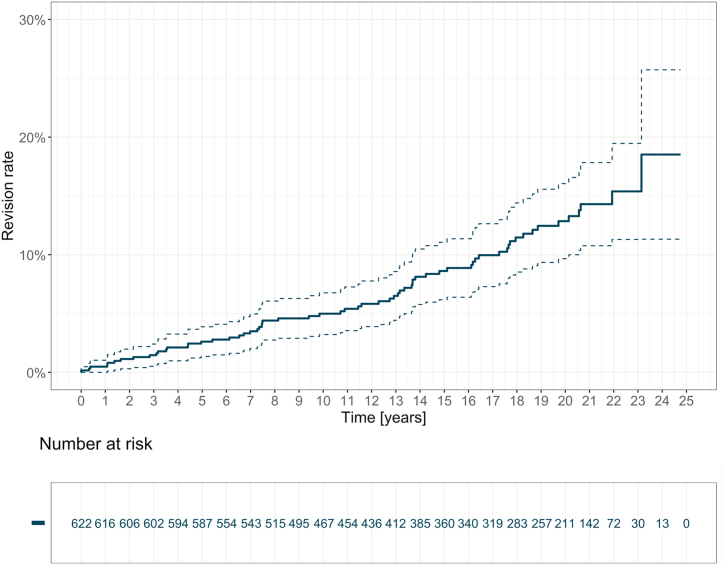
Overall revision rate. Kaplan-Meier estimate of the cumulative revision rate over time of the entire cohort. The solid line represents the revision rate, with dashed lines indicating the 95% confidence intervals. The number of cases at risk is indicated in the table below the curve.

**Figure 4:**
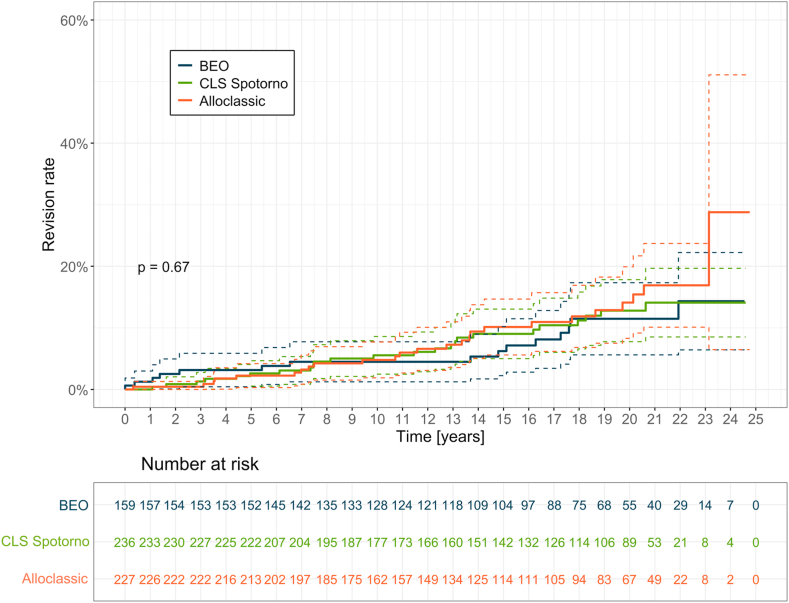
Revision rate by stem type. Kaplan-Meier estimate of the revision rates observed in association with the BEO (black), CLS Spotorno (green), and Alloclassic (orange) stems. The dashed lines represent the 95% confidence intervals. The *P* value of .67 indicates no significant difference in revision rates between the groups. The number of cases at risk each year is indicated in the table below the figure.

**Figure 5:**
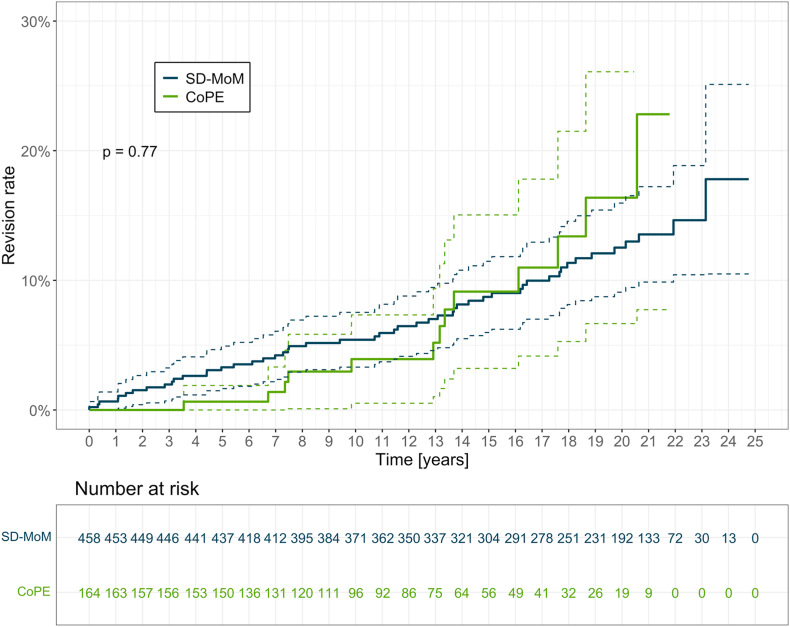
Revision rate by bearing type. Kaplan-Meier estimate of the revision rates observed in association with the SD-MoM bearing (blue) and the CoPE bearing (green). Dashed lines represent the 95% confidence intervals. The *P* value of .44 indicates no significant difference in revision rates between the groups. However, there were differences in the pattern. While the revision curve for the SD-MoM bearing grows linearly, the revision rate of the CoPE bearing was initially very low and showed a steeper increase over the second decade. The number of cases at risk at each time point is indicated in the table below figure.

The global revision rate increased almost linearly over time for the entire cohort (Fig. 3). No significant differences were found in long-term revision rates depending on the stem used (BEO vs CLS Spotorno vs Alloclassic: *P* = .67; BEO & CLS Spotorno vs Alloclassic: *P* = .42) (Fig. 4). There also was no difference within the subgroup of SD-MoM (*P* = .59). As depicted in Figure 5, SD-MoM bearings exhibited a constant increase in revision rates over time, maintaining a linear progression without a notable spike even by 20 years of follow-up. In contrast, no revisions were observed during the first 2 years for cases with CoPE bearings. However, the revision rates of this subgroup rose more sharply after 10 years, surpassing SD-MoM at 15 years with 9% and peaking at 16% by 20 years. However, this difference was not statistically significant (*P* = .77). Notably, only 28-mm heads were used throughout the cohort. Detailed values are listed in Table 2.

**Table 2:** Revision rates.

Revision rate % (95% CI)	1 year	2 years	5 years	10 years	15 years	20 years	24 years
Overall	0.5 (0.0-1.0)	1.1 (0.3-2.0)	2.6 (1.4-3.9)	5.0 (3.2-7.5)	8.6 (6.2-11.1)	12.9 (9.7-16.0)	18.5 (11.3-25.7)
Stem							
BEO	1.3 (0.0-3.0)	2.5 (0.1-5.0)	3.2 (0.4-5.9)	4.5 (1.2-7.7)	6.2 (2.2-10.2)	11.5 (5.6-17.3)	14.3 (6.4-22.2)
CLS Spotorno	0 (0.0-0.0)	0.9 (0.0-2.1)	2.6 (0.6-4.7)	5.5 (2.5-8.6)	9.0 (5.0-13.0)	12.8 (7.8-17.8)	14.1 (8.5-19.7)
Alloclassic	0.4 (0.0-1.3)	0.4 (0.0-1.3)	2.2 (0.3-4.2)	4.8 (1.9-7.7)	10.1 (5.6-14.7)	14.1 (8.3-19.9)	28.8 (6.5-51.1)
CLS Spotorno and BEO	0.5 (0.0-1.2)	1.5 (0.3-2.7)	2.8 (1.2-4.5)	5.1 (2.9-7.3)	7.8 (5.0-10.7)	12.2 (8.4-16.1)	14.6 (9.5-19.6)
Bearing							
SD-MoM	0.7 (0.0-1.4)	1.5 (0.4-2.7)	3.3 (1.7-4.9)	5.4 (3.3-7.5)	8.7 (6.0-11.5)	12.5 (9.1-16.0)	17.8 (10.5-25.1)
CoPE	0 (0.0-0.0)	0 (0.0-0.0)	0.6 (0.0-1.9)	3.9 (0.5-7.3)	9.1 (3.2-15.1)	16.4 (6.7-26.1)	n/a

This table provides the Kaplan-Meier estimate of the revision rate over time. Percentages with 95% confidence intervals are indicated. Results are listed for the entire cohort and subgrouped by stem, respectively, by bearing.

N/a, not available.

To account for differences in age and sex characteristics, a multivariate Cox proportional hazards model was applied including age and sex as covariates. After adjusting, no significant association between bearing type and risk of revision was observed (HR for SD-MoM vs CoPE: 1.37; 95% CI: 0.65-2.91; *P* = .409). Age was a significant predictor of revision (HR per year increase: 0.97; 95% CI 0.94-0.999; *P* = .043), while sex was not (HR for male vs female: 1.06; 95% CI: 0.62-1.81; *P* = .834).

**Figure 6:**
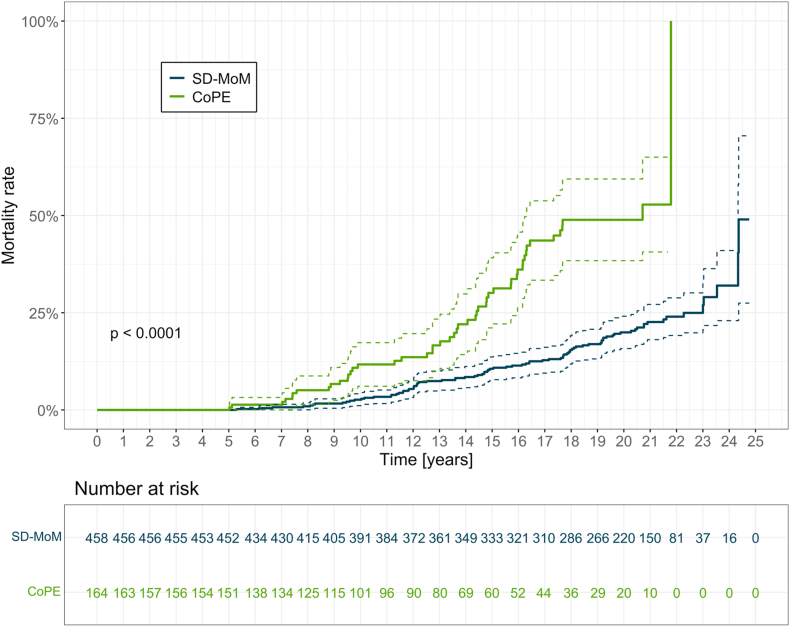
Mortality rate by bearing type. Kaplan-Meier survival analysis illustrating the cumulative mortality rates for patients with SD-MoM (blue) vs CoPE (green) bearings. The solid lines represent the estimated mortality rate for each group. The dashed lines indicate the 95% confidence intervals. The *P* value <.0001 denotes a statistically significant difference between the 2 groups. Note the age difference between both groups (median 61.9 years for SD-MoM vs median 74.2 years for CoPE, *P* < .001), which is the most probable explanation.

Mortality rates differed significantly between SD-MoM and CoPE bearings (Fig. 6), being higher for the latter (*P* < .0001). At 10 years, mortality was 3% for SD-MoM and 12% for CoPE, increasing to 20% and 49% at 20 years, respectively (details in Table 3).

**Table 3:** Mortality rate.

Mortality rate % (95% CI)	1 year	2 years	5 years	10 years	15 years	20 years	24 years
Overall	0.0 (0.0-0.0)	0.0 (0.0-0.0)	0.0 (0.0-0.0)	4.7 (3.0-6.5)	14.6 (11.5-17.7)	25.8 (21.7-29.8)	37.7 (29.3-46.2)
SD-MoM	0.0 (0.0-0.0)	0.0 (0.0-0.0)	0.0 (0.0-0.0)	2.6 (1.1-4.2)	10.6 (7.6-13.6)	20.0 (15.8-24.1)	32.0 (23.0-41.0)
CoPE	0.0 (0.0-0.0)	0.0 (0.0-0.0)	0.0 (0.0-0.0)	11.7 (6.1-17.3)	30.1 (21.1-39.1)	48.9 (38.4-59.4)	n/a

This table provides the mortality rate over time of the entire cohort, respectively, differentiated by bearing (SD-MoM vs CoPE). Estimated mean and 95% confidence intervals are indicated.

N/a, not available.

In the Cox model for mortality, CoPE was associated with increased risk after adjusting for age and sex (HR: 1.20; 95% CI: 1.12-1.91; *P* = .002). Age remained a strong predictor of mortality risk (HR per year: 1.12; 95% CI: 1.09-1.16; *P* < .001), and females had lower mortality (HR: 0.43; 95% CI: 0.29-0.61; *P* < .001).

## Discussion

This retrospective study provided some specific insights into long-term outcomes after uncemented THA with different stem designs and bearing couples. The overall low revision rate was comparable to previous reports. The various implants used in this cohort had no statistically significant influence on the revision rates over time. However, a different pattern of cumulative revision rates over time was observed for SD-MoM and CoPE. No increased mortality could be observed for SD-MoM bearings, but this observation was biased by a largely favorable patient selection.

Outcomes up to 20 years or more are reported only rarely from single centers [[10], [11], [12], [13],^16^,^24^,^25^]. In our study, consistency in the surgical technique during the recruitment period allowed some valuable comparisons. More specifically, this study is one of the largest cohorts of SD-MoM analyzed on the long term [13,16].

**Figure 7:**
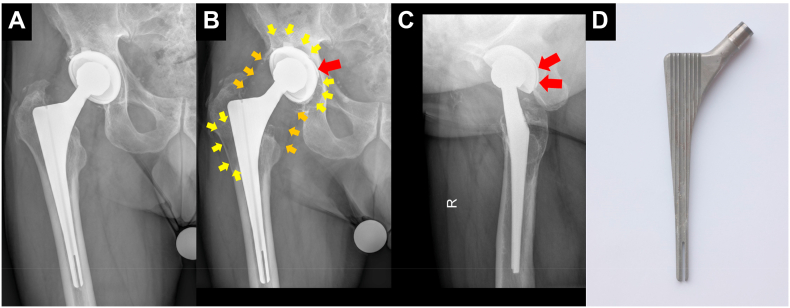
BEO stem. Radiographs and picture of a retrieval illustrating a BEO stem. In (a), zone of interest of an anteroposterior radiograph of the pelvis 10 years after uncemented total hip arthroplasty (THA) with a Fitmore cup with a metal-polyethylene-sandwich inlay type Metasul 28 mm and a BEO stem. In (b), the last radiograph before revision, 15 years after the primary THA, with the corresponding axial view in (c). Note the change of orientation of the loose cup and delamination of the Sulmesh coating (red arrows). Yellow arrows mark osteolysis in the acetabulum as well as the proximal femur. The wear particle reaction also formed a pseudotumor visible radiographically (orange arrows). In (d), picture of the retrieved stem. Note the similarities to a CLS Spotorno stem the BEO evolved from. The BEO has longitudinal ribs extending along the whole length of the anterior and posterior surface, is cannulated, as visible on the radiographs, and its tip is slotted to reduce stiffness.

Our study showed a consistent linear increase of the global revision rate up to 24 years, with only a slight rise after more than 10 years. Usually, after the revisions caused during the first months by intraoperative and early postoperative complications, the revision rate should only increase very slowly up to 10 years of follow-up, after which revisions due to long-term issues such as wear and periprosthetic fractures predominate [17,26]. While the usual pattern of distribution over time was not observed, the revision rates at 5 years (2.6%), 10 years (5%), 15 years (8.6%), and at 20 years (12.9%) align with data from national registries and other individual studies (Figure 4, Figure 5, Figure 6, Figure 7) [8,13,17,24,[27], [28], [29], [30], [31], [32]]. This agreement reinforces the findings of this study, despite limitations of registry studies such as the lack of reported bearing couple types and disregarding the effect of healthcare access on absolute revision rates. Differences among national registries are illustrated by a much lower revision burden in the UK than in other countries [17,26,28,29,31], which may in part be explained by the relatively more frequent use of cemented stems in the UK, which is, however, similar to the Scandinavian countries [29,31]. However, with 5% revision rate at 10 years, the results of this cohort would barely meet the benchmark defined by the UK national healthcare institutions [6].

No significant differences in revision rates were observed among the different stem types (BEO vs CLS Spotorno vs Alloclassic; *P* = .67), with all showing similar survival patterns (Fig. 4). At 20-year follow-up, the Kaplan-Meier estimate of the revision rate of THA with the BEO, CLS Spotorno, and the Alloclasic stem was 11.5%, 12.8%, and 14.1%, respectively. The BEO stem—named by its designers after the radio station Berner Oberland in Switzerland—was a variant of the CLS Spotorno that is no longer available. The BEO stem has extended longitudinal ribs, is cannulated, and has a slotted tip, which was designed with the intend to reduce thigh pain (Fig. 7). Otherwise, both stems are identical, which is supported by the similar outcomes observed in this study (Table 3 and Fig. 4). To the best of our knowledge, this study would however be the first report of long-term results of the BEO stem, beyond a case report [33].

**Figure 8:**
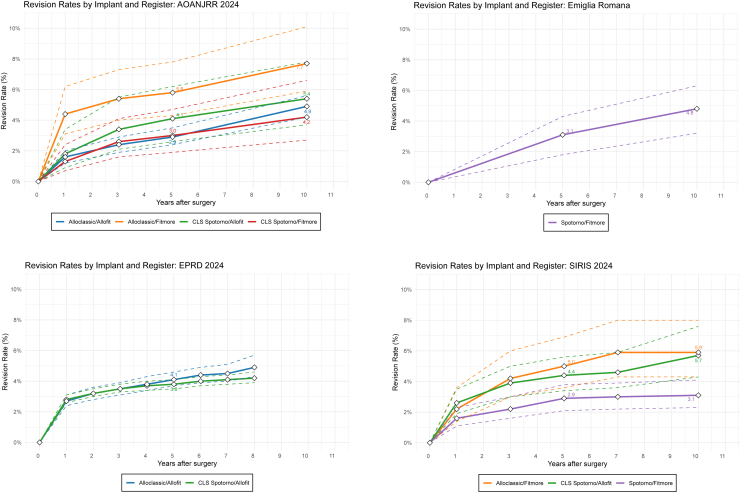
Revision rates of national registries. Summary of the global revision rates extracted from the annual reports of the national registries of Australia (top left), Emilia-Romania (top right), Germany (bottom left), and Switzerland (bottom right), for THA with the following stem/cup combinations: Alloclassic Allofit, Alloclassic/Fitmore, CLS Spotorno/Allofit, and CLS Spotorno/Fitmore. Note the difference in follow-up available in the various registries. Only a relative comparison within each registry is possible. Cross-registry comparisons are not correct due to influences by differences in healthcare system and availability of revision THA.

Some studies and registries reported relatively low revision rates for CLS Spotorno of 2.9% to 6.2% at 15 years [28,34,35]. Others reported higher revision rates, including the Finnish registry, with 34% at 15 years, and Evola et al. with 19.7% at 23 years [11,32]. For the Alloclassic stem, the Australian registry reported comparable results at 15 years, with an 8.3% revision rate [28]. Lower revision rates were observed by Suckel et al. at 4.8% over 17 years, while Pisecky et al. documented 11% at 30 years, and Andeol et al. reported 9.3% at 18.6 years [10,12,36]. An overview of global revision rates, for combinations with cups used also in this cohort, extracted from the annual reports of various national arthroplasty registries is provided in Figure 8. However, long-term revision rates are highly dependent on wear. Therefore, the bearing surface design and material increase in relevance in the second decade in situ onward, likely even more than the design of the stem. Yet, information about the bearing couple is usually not reported, particularly in registries. Of note, individual studies tend to report better long-term results than national registries, a phenomenon identified specifically for the CLS Spotorno stem, which is also among the implants in our study [37].

While revision rates between CoPE and SD-MoM bearings were not significantly different overall (*P* = .44), SD-MoM showed a steady increase, while CoPE exhibited an accelerated increase beyond 10 years after initially very low revision rates (Fig. 5). Increasing age is associated with decreasing revision rates over time [29,31], and the CoPE group was significantly older than the SD-MoM group (median 74.2 vs 61.9 years, *P* < .001), similar to a recent report from another center in Switzerland [13]. However, the cumulative revision rate observed during the first years was surprisingly low in the CoPE group (Fig. 5). This finding may be explainable by a random effect due to relatively small numbers.

At the time of their introduction, SD-MoM bearings were considered a favorable alternative to conventional polyethylene, particularly due to dramatically reduced volumetric wear rates [38]. These attributes made SD-MoM an attractive option, particularly for younger, more active patients, as they promised greater implant longevity. Therefore, the adoption of SD-MoM bearings was a logical and justifiable choice at the time [13,[38], [39], [40], [41]]. Some studies reported more favorable outcomes with SD-MoM compared to CoPE, particularly in younger patients [40,41]. These studies should, however, be viewed with caution, as the CoPE group did not benefit from modern prosthetic designs (Harris-Galante I cup with conventional polyethylene, ABG I stem) [40]. A few studies reported acceptable revision rates for SD-MoM ranging from 3.9 to 6% at 10 to 15 years [[42], [43], [44], [45]]. Other studies reported higher revision rates of 8 to 13% over 13 to 20 years of follow-up after THA with SD-MoM bearing [16,[46], [47], [48], [49]]. However, a recent study with a study cohort comparable to ours identified higher long-term revision rates for SD-MoM than for CoPE [13]. With the emergence of highly cross-linked polyethylene (HXLPE), the wear rate was dramatically reduced compared to conventional polyethylene, making MoM bearings obsolete [50,51]. While our study is limited by the numbers available, with no statistically significant difference between the bearings (*P* = .44), a clear midterm increase in revision rate was observed in the second decade after THA with CoPE (Fig. 5), as would be expected for such a bearing. In summary, SD-MoM bearings show variable long-term performance, with some favorable outcomes in younger patients, but generally poorer results compared to bearings with HXLPE sockets [27,28,31]. The main reasons for failure of large-diameter MoM are design-specific low cup articular arc angle (below 180°) and radial clearances, leading to a higher risk of edge loading. Edge loading is associated with higher metal wear rates, as well as the release of larger cobalt-chromium-molybdenum particles that are more likely to cause adverse local tissue reactions. Additionally, higher friction torques associated with larger head diameter are considered a risk factor for the onset of fretting corrosion at the taper junction, and thus the risk of trunnionosis [[52], [53], [54]]. All these issues are not common in SD-MoM. Nowadays, registry data and clinical studies demonstrate a preference for HXLPE bearings over MoM bearing [27,30,31,33,55]. The evolution of technology made the issue irrelevant.

As a meta-analysis analyzing randomized trials of stemmed MoM and resurfacing arthroplasty, had identified a higher mortality risk beyond 10 years for recipients of MoM THA, with an 8.5% increased risk, suggesting a potential dose-response relationship with prolonged exposure to metal wear debris [56]. In our study, however, no late increase of mortality was observed in the SD-MoM group. In fact, mortality rates were significantly higher for CoPE than for SD-MoM at 20 years (49% vs 20%, *P* < .0001), which can be explained by the younger age of the SD-MoM group. No detailed comparison to the national population was possible, as publicly available data do not allow appropriate matching. Gonzalez et al. conducted a large cohort study and reported findings consistent with our results, demonstrating substantially higher mortality in the CoPE group. They presented mortality for the entire 26.8-year observation period, with 34.7% deaths in the MoM group and 70.6% in the CoPE group. These outcomes align with our data, showing 32% mortality in the SD-MoM group at 24 years and 48.9% mortality at 20 years in the CoPE group. The authors proposed that this was because second-generation MoM prostheses were considered superior to CoPE at the time, resulting in younger patients being more likely to receive MoM implants. After adjustment for confounders, they found that the HR for MoM was even slightly reduced [13]. Mortality after primary THA is reported ranging from 55 to 60% at up to 20 years of follow-up [28,31]. Therefore, the significant age disparity between groups in our study (median 62 years for SD-MoM compared to 74 years for CoPE) likely accounts for the observed differences in mortality rates. This assumption would be supported by data from the UK National Joint Registry, which shows that mortality risk more than doubles between these age groups [31]. Kurtz et al. reported a 6.1% mortality rate for CoPE bearings at 8 years, lower than our 11.7% at 10 years [57].

The study's primary strength is reporting follow-up of at least 20 years and up to 24 years, offering valuable insights into the long-term performance of uncemented THA. Subgroup analysis regarding bearing and stem types could also be performed. However, the retrospective design of the study introduced inherent limitations, such as possibly incomplete identification of revisions, selection bias with exclusion of patients who did not undergo revision despite experiencing complications requiring reoperation, such as internal fixation of periprosthetic fractures and limited possibilities for identification of confounding factors. Periprosthetic fractures are a complication particularly affecting uncemented THA early, but also with increasing long-term risk [14,58,59]. This limitation may result in an underestimation of potentially life-threatening complications and true reoperation rates, as patients with prosthetic failures who were treated with internal fixation instead of revision may have been missed. A significant limitation is the age disparity between the groups, the SD-MoM group being significantly younger than the CoPE group (61.9 vs 74.2 years, *P* < .001). Furthermore, the study could not control systematically for critical patient-related factors, such as body mass index, comorbidities, and activity level, which are known to impact revision and mortality rates [[28], [29], [30], [31],^60^]. Despite these limitations, the long-term data provide valuable insights, while emphasizing the need for cautious interpretation.

## Conclusions

Long-term revision rates of uncemented THA in this cohort were comparable to other reports, but single-center studies with such long follow-up remain rare. There were no statistically significant differences in revision rates among the various implants used, but clear differences in the evolution of cumulative revision rates over time were observed for SD-MoM vs CoPE. While SD-MoM bearings were once the best solution available in THA, having been introduced about a decade before HXLPE became available, very recent data suggest complications emerging after 10 to 20 years in situ. SD-MoM are nevertheless associated with low long-term revision rates, in contrast to large diameter MoM, which are associate with overall higher revision rates, adverse tissue responses, and systemic effects, resulting in the fast decline of MoM bearing in THA. Mortality appeared significantly lower in the SD-MoM group compared to the CoPE group, mostly by age differences favoring the former. However, no late increase of mortality was observed in the SD-MoM group, which may have been indicative of toxicity issues. Ongoing data collection and further analysis are necessary to establish and refine guidelines for clinical use.

## CRediT authorship contribution statement

**Sandro Beros:** Writing – review & editing, Writing – original draft, Data curation. **Philemon Grimm:** Investigation, Data curation. **Michel Schläppi:** Formal analysis, Data curation. **Robin Pourzal:** Writing – review & editing. **Joshua J. Jacobs:** Writing – review & editing. **Peter Wahl:** Writing – review & editing, Writing – original draft, Investigation, Conceptualization.

## Conflicts of interest

J.J. Jacobs receives royalties from Signature Orthopaedics; is a paid consultant for Academic Orthopaedic Consortium; has stock or stock options in Hyalex and MedTex Polaris; receives royalties, financial, or material support from JBJS Board of Trustees; is a member of the medical/orthopaedic publications editorial/governing board at JBJS Board of Trustees; and is a board member/committee appointments for OREF. P. WAHL is a member of the Expert Group Infection of Swiss Orthopaedics and is a member of the Expert Group of the Swiss Arthroplasty Registry SIRIS. R. Pourzal is on the speakers bureau/paid presentations for CeramTec at the Annual Meeting of ISTA, 2025; and receives research support from Zimmer Biomet and Enovis; all other authors declare no potential conflicts of interest.

For full disclosure statements refer to https://doi.org/10.1016/j.artd.2026.102078.

## Appendix A Supplementary data

Conflict of Interest Statement for Schläppi

Conflict of Interest Statement for Jacobs

Conflict of Interest Statement for Wahl

Conflict of Interest Statement for Beros

Conflict of Interest Statement for Grimm

Conflict of Interest Statement for Pourzal

## References

[1] George L.K., Ruiz D., Sloan F.A. (2008). The effects of total hip arthroplasty on physical functioning in the older population. J Am Geriatr Soc.

[2] Hannon C.P., Goodman S.M., Austin M.S., Yates A., Guyatt G., Aggarwal V.K. (2023). 2023 American college of rheumatology and American Association of hip and Knee Surgeons Clinical Practice Guideline for the optimal timing of elective hip or knee arthroplasty for patients with symptomatic moderate-to-severe osteoarthritis or advanced symptomatic osteonecrosis with secondary arthritis for whom nonoperative therapy is ineffective. J Arthroplasty.

[3] Saleem A., Lin C.C., Anil U., Rivero S.M. (2024). Arthroplasty treatment options for femoral neck fractures in the elderly: a network meta-analysis of randomized control trials. Injury.

[4] Roth A., Beckmann J., Bohndorf K., Fischer A., Heiß C., Kenn W. (2016). S3-Guideline non-traumatic adult femoral head necrosis. Arch Orthop Trauma Surg.

[5] Strahl A., Kazim M.A., Kattwinkel N., Hauskeller W., Moritz S., Arlt S. (2022). Mid-term improvement of cognitive performance after total hip arthroplasty in patients with osteoarthritis of the hip : a prospective cohort study. Bone Joint J.

[6] Kandala N.B., Connock M., Pulikottil-Jacob R., Sutcliffe P., Crowther M.J., Grove A. (2015). Setting benchmark revision rates for total hip replacement: analysis of registry evidence. BMJ.

[7] Sadoghi P., Schroder C., Fottner A., Steinbruck A., Betz O., Muller P.E. (2012). Application and survival curve of total hip arthroplasties: a systematic comparative analysis using worldwide hip arthroplasty registers. Int Orthop.

[8] Evans J.T., Evans J.P., Walker R.W., Blom A.W., Whitehouse M.R., Sayers A. (2019). How long does a hip replacement last? A systematic review and meta-analysis of case series and national registry reports with more than 15 years of follow-up. Lancet.

[9] Nugent M., Young S.W., Frampton C.M., Hooper G.J. (2021). The lifetime risk of revision following total hip arthroplasty. Bone Joint J.

[10] Andeol Q., Viste A., Desmarchelier R., Lerat J.L., Fessy M.H. (2020). Metasul vs Cerasul bearings: a prospective, randomized study at a mean eighteen years. Int Orthop.

[11] Evola F.R., Evola G., Graceffa A., Sessa A., Pavone V., Costarella L. (2014). Performance of the CLS Spotorno uncemented stem in the third decade after implantation. Bone Joint J.

[12] Pisecky L., Hipmair G., Schauer B., Böhler N. (2018). 30-years of experience with the cementless implanted Alloclassic total hip arthroplasty system-An ultra-long-term follow-up. J Orthop.

[13] Gonzalez A.I., Barea C., Zingg M., Garavaglia G., Peter R., Hoffmeyer P. (2025). Long-term outcomes of small head metal-on-metal compared to ceramic-on-polyethylene primary total hip arthroplasty: a registry-based cohort study. Int Orthop.

[14] Konow T., Baetz J., Melsheimer O., Grimberg A., Morlock M. (2021). Factors influencing periprosthetic femoral fracture risk. Bone Joint J.

[15] Jasty M., Maloney W.J., Bragdon C.R., O'Connor D.O., Haire T., Harris W.H. (1991). The initiation of failure in cemented femoral components of hip arthroplasties. J Bone Joint Surg Br.

[16] de Steiger R., Peng A., Lewis P., Graves S. (2018). What is the long-term survival for primary THA with small-head metal-on-metal bearings?. Clin Orthop Relat Res.

[17] Swiss-Registry Annual report of the SIRIS registry hip and knee 2023. https://www.swiss-medtech.ch/sites/default/files/2023-11/231117_SIRIS-Report-2023_Final_online.pdf.

[18] Kendal A.R., Prieto-Alhambra D., Arden N.K., Carr A., Judge A. (2013). Mortality rates at 10 years after metal-on-metal hip resurfacing compared with total hip replacement in England: retrospective cohort analysis of hospital episode statistics. BMJ.

[19] Bradberry S.M., Wilkinson J.M., Ferner R.E. (2014). Systemic toxicity related to metal hip prostheses. Clin Toxicol (Phila).

[20] Cheung A.C., Banerjee S., Cherian J.J., Wong F., Butany J., Gilbert C. (2016). Systemic cobalt toxicity from total hip arthroplasties: review of a rare condition Part 1 - history, mechanism, measurements, and pathophysiology. Bone Joint J.

[21] Leyssens L., Vinck B., Van Der Straeten C., Wuyts F., Maes L. (2017). Cobalt toxicity in humans—A review of the potential sources and systemic health effects. Toxicology.

[22] Swiatkowska I., Henckel J., Sabah S.A., Hart A.J. (2022). Self-reported neurotoxic symptoms in hip arthroplasty patients with highly elevated blood cobalt: a case-control study. J Patient Saf.

[23] Bauer R., Kerschbaumer F., Poisel S., Oberthaler W. (1979). The transgluteal approach to the hip joint. Arch Orthop Trauma Surg (1978).

[24] Bayliss L.E., Culliford D., Monk A.P., Glyn-Jones S., Prieto-Alhambra D., Judge A. (2017). The effect of patient age at intervention on risk of implant revision after total replacement of the hip or knee: a population-based cohort study. Lancet.

[25] Rizzo M., Balato G., Cerbasi S., Costa G., Guarino A., Mariconda M. (2020). Long-Term survival and results at a mean Follow-Up period of 24 years of a tapered straight, collarless, Grit-Blasted, titanium alloy stem. J Arthroplasty.

[26] Grimberg A., Lützner J., Melsheimer O., Morlock M., Steinbrück A. (2023). Deutsche Gesellschaft für Orthopädie und orthopädische Chirurgie.

[27] Registry N.J. The New Zealand Joint Registry 2023. https://www.nzoa.org.nz/sites/default/files/NZJR%20Twenty%20Four%20Year%20Report__29Aug2023.pdf.

[28] Lewis P.L.G.D., McAuliffe M.J., McDougall C., Stoney J.D., Vertullo C.J., Wall C.J. (2024).

[29] Rolfson AW-DJKCRJNEBPIAMMO Annual report 2023: the Swedish Arthroplasty Register. https://registercentrum.blob.core.windows.net/sar/r/SAR_Annual-report-2023_EN-DS5gryeOB.pdf.

[30] R.I.P.O (2020). Report of RIPO: Regional Register of Orthopaedic Prosthetic Implantology - Overall data on hip, knee and shoulder arthroplasty in Emilia-Romagna Region (2000-2020).

[31] Achakri H., Ben-Shlomo Y., Blom A., Boulton C., Bridgens J., Brittain R., Clark E. (2022).

[32] Register F.A. (2023). Annual Counts 2023: National Institute for Health an Welfare (THL). https://www2.thl.fi/endo/report/#data/hip_rev_years.

[33] Sutter L., Hall D.J., Bischoff L., Dommann-Scherrer C., Schläppi M., Pourzal R. (2025). How in vivo alteration of hip replacement wear mode can cause a voluminous inflammatory reaction and an excessive titanium exposure. J Clin Med.

[34] Registry D.H.A. Danish Hip Arthroplasty Registry, National Annual Report for 2021. https://saks.ortopaedi.dk/wp-content/uploads/2022/09/DHAR_Annual_report_2021.pdf.

[35] Dhillon M.S., Jindal K., Kumar P., Rajnish R.K., Neradi D. (2022). Long-term survival of CLS Spotorno femoral stem: a systematic review of literature. Arch Orthop Trauma Surg.

[36] Suckel A., Geiger F., Kinzl L., Wulker N., Garbrecht M. (2009). Long-term results for the uncemented Zweymuller/Alloclassic hip endoprosthesis. A 15-year minimum follow-up of 320 hip operations. J Arthroplasty.

[37] Sadoghi P., Janda W., Agreiter M., Rauf R., Leithner A., Labek G. (2013). Pooled outcome of total hip arthroplasty with the CementLess Spotorno (CLS) system: a comparative analysis of clinical studies and worldwide arthroplasty register data. Int Orthop.

[38] Sieber H.P., Rieker C.B., Kottig P. (1999). Analysis of 118 second-generation metal-on-metal retrieved hip implants. J Bone Joint Surg Br.

[39] GmbH Z. (2009).

[40] Migaud H., Putman S., Krantz N., Vasseur L., Girard J. (2011). Cementless metal-on-metal versus ceramic-on-polyethylene hip arthroplasty in patients less than fifty years of age: a comparative study with twelve to fourteen-year follow-up. J Bone Joint Surg Am.

[41] Grélier M., Martinot P., Dartus J., Migaud H., Putman S., Girard J. (2023). Cementless metal-on-metal versus ceramic-on-polyethylene hip arthroplasty in under-50 year-olds with 20 to 22 years' follow-up: was it a good idea to abandon the small-diameter metal-on-metal bearing?. Orthop Traumatol Surg Res.

[42] Delaunay C.P., Putman S., Puliéro B., Bégin M., Migaud H., Bonnomet F. (2016). Cementless total hip arthroplasty with metasul bearings provides good results in active young patients: a concise followup. Clin Orthop Relat Res.

[43] Randelli F., Banci L., D'Anna A., Visentin O., Randelli G. (2012). Cementless Metasul metal-on-metal total hip arthroplasties at 13 years. J Arthroplasty.

[44] Zuiderbaan H.A., Visser D., Sierevelt I.N., Penders J., Verhart J., Vergroesen D.A. (2018). Long-term clinical results of the Metasul metal-on-metal total hip arthroplasty: 12.6 years follow-up of 128 primary total hip replacements. Hip Int.

[45] Zijlstra W.P., van Raay J.J., Bulstra S.K., Deutman R. (2010). No superiority of cemented metal-on-metal over metal-on-polyethylene THA in a randomized controlled trial at 10-year follow-up. Orthopedics.

[46] Tardy N., Maqdes A., Boisrenoult P., Beaufils P., Oger P. (2015). Small diameter metal-on-metal total hip arthroplasty at 13 years - a follow-up study. Orthop Traumatol Surg Res.

[47] Malek I.A., Rao S.P., Rath N.K., Mallya U.N. (2015). Cemented metal-on-metal total hip replacement with 28-mm head: prospective, long-term, clinical, radiological and metal ions data. Eur J Orthop Surg Traumatol.

[48] Lass R., Grübl A., Kolb A., Domayer S., Csuk C., Kubista B. (2014). Primary cementless total hip arthroplasty with second-generation metal-on-metal bearings: a concise follow-up, at a minimum of seventeen years, of a previous report. J Bone Joint Surg Am.

[49] Erivan R., Villatte G., Millerioux S., Mulliez A., Descamps S., Boisgard S. (2020). Survival at 11 to 21 years for 779 metasul® metal-on-metal total hip arthroplasties. J Orthop Surg (Hong Kong).

[50] GmbH Z. (2006).

[51] de Steiger R., Lorimer M., Graves S.E. (2018). Cross-Linked polyethylene for total hip arthroplasty markedly reduces revision surgery at 16 years. J Bone Joint Surg Am.

[52] Matthies A., Underwood R., Cann P., Ilo K., Nawaz Z., Skinner J. (2011). Retrieval analysis of 240 metal-on-metal hip components, comparing modular total hip replacement with hip resurfacing. J Bone Joint Surg Br.

[53] Rieker C.B., Wahl P. (2020). What the surgeon can Do to reduce the risk of trunnionosis in hip arthroplasty: recommendations from the literature. Materials (Basel).

[54] Underwood R.J., Zografos A., Sayles R.S., Hart A., Cann P. (2012). Edge loading in metal-on-metal hips: low clearance is a new risk factor. Proc Inst Mech Eng H.

[55] Marchetti E., Krantz N., Berton C., Bocquet D., Fouilleron N., Migaud H. (2011). Component impingement in total hip arthroplasty: frequency and risk factors. A continuous retrieval analysis series of 416 cup. Orthop Traumatol Surg Res.

[56] Pijls B.G., Meessen J.M., Schoones J.W., Fiocco M., van der Heide H.J., Sedrakyan A. (2016). Increased mortality in metal-on-metal versus non-metal-on-metal primary total hip arthroplasty at 10 years and longer Follow-Up: a systematic review and meta-analysis. PLoS One.

[57] Kurtz S.M., Lau E., Baykal D., Springer B.D. (2017). Outcomes of ceramic bearings after primary total hip arthroplasty in the Medicare population. J Arthroplasty.

[58] Kelly M., Chen A.F., Ryan S.P., Working Z.M., Porter K.R., De A. (2023). Cemented femoral fixation in total hip arthroplasty reduces the risk of periprosthetic femur fracture in patients 65 years and older: an analysis from the American joint replacement registry. J Arthroplasty.

[59] Lewis S.R., Macey R., Parker M.J., Cook J.A., Griffin X.L. (2022). Arthroplasties for hip fracture in adults. Cochrane Database Syst Rev.

[60] Schmalzried T.P., Shepherd E.F., Dorey F.J., Jackson W.O., dela Rosa M., Fa'vae F. (2000). The John Charnley Award. Wear is a function of use, not time. Clin Orthop Relat Res.

