# Supplementary material for: Survival in the Third Decade of Uncemented Total Hip Arthroplasty With Small-Diameter Metal-on-Metal Versus Ceramic-on-Conventional Polyethylene Bearings
Source: Arthroplast Today. 2026 Jul 8;40:102078. doi: 10.1016/j.artd.2026.102078 (PMC13380179; doi:10.1016/j.artd.2026.102078)
Supplement: Conflict of Interest Statement for Wahl [file mmc3.pdf]

CONFLICT OF INTEREST STATEMENT

American Association of Hip and Knee Surgeons

(Adopted from the American Academy of Orthopaedic Surgeons disclosure statement)

The following form **must be filled out completely and submitted by each author (example, 6 authors, 6 forms).**
All items require a response. If there is no relevant disclosure for a given item, enter "*None.*"

20 TO 24 YEARS SURVIVAL AFTER UNCEMENTED TOTAL HIP ARTHROPLASTY WITH SMALL-DIAMETER METAL-ON-METAL OR WITH CERAMIC-ON-CONVENTIONAL POLYETHYLENE BEARINGS

1. Royalties from a company or supplier (The following conflicts were disclosed)

   none

2. Speakers bureau/paid presentations for a company or supplier (The following conflicts were disclosed)

   none

3A. Paid employee for a company or supplier (The following conflicts were disclosed)

   none

3B. Paid consultant for a company or supplier (The following conflicts were disclosed)

   none

3C. Unpaid consultants for a company or supplier (The following conflicts were disclosed)

   none

4. Stock or stock options in a company or supplier (The following conflicts were disclosed)

   none

5. Research support from a company or supplier as a Principal Investigator (The following conflicts were disclosed)

   none

6. Other financial or material support from a company or supplier (The following conflicts were disclosed)

   none

7. Royalties, financial or material support from publishers (The following conflicts were disclosed)

   none

8. Medical/Orthopaedic publications editorial/governing board (The following conflicts were disclosed)

   nonw

9. Board member/committee appointments for a society (The following conflicts were disclosed)

   Member of the Expert Group Infection of Swiss Orthopaedics
   Member of the Expert Group of the Swiss Arthroplasty Registry SIRIS

Each author must sign AND print or type his/her name, date and submit a separate form
In addition, one BLINDED Conflict of Interest form (no author names used) should be submitted per manuscript with all author disclosures.

Peter WAHL	P. Wahl	25.02.2026
Author Name (Print or Type)	Author Signature	Date
