# Supplementary material for: Survival in the Third Decade of Uncemented Total Hip Arthroplasty With Small-Diameter Metal-on-Metal Versus Ceramic-on-Conventional Polyethylene Bearings
Source: Arthroplast Today. 2026 Jul 8;40:102078. doi: 10.1016/j.artd.2026.102078 (PMC13380179; doi:10.1016/j.artd.2026.102078)
Supplement: Conflict of Interest Statement for Beros [file mmc4.pdf]

BLINDED **CONFLICT OF INTEREST STATEMENT**

American Association of Hip and Knee Surgeons

(Adopted from the American Academy of Orthopaedic Surgeons disclosure statement)

The following form **must be filled out completely listing all author affiliations**. **All items require a response. If there is no relevant disclosure for a given item, enter "*None*."**

Manuscript Title

1. Royalties from a company or supplier (The following conflicts were disclosed)
   None

2. Speakers bureau/paid presentations for a company or supplier (The following conflicts were disclosed)
   None

3A. Paid employee for a company or supplier (The following conflicts were disclosed)
   None

3B. Paid consultant for a company or supplier (The following conflicts were disclosed)
   None

3C. Unpaid consultants for a company or supplier (The following conflicts were disclosed)
   None

4. Stock or stock options in a company or supplier (The following conflicts were disclosed)

   None

5. Research support from a company or supplier as a Principal Investigator (The following conflicts were disclosed)

   None

6. Other financial or material support from a company or supplier (The following conflicts were disclosed)

   None

7. Royalties, financial or material support from publishers (The following conflicts were disclosed)

   None

8. Medical/Orthopaedic publications editorial/governing board (The following conflicts were disclosed)

   None

9. Board member/committee appointments for a society (The following conflicts were disclosed)

   None

In addition, one BLINDED Conflict of Interest form (no author names used) should be submitted per manuscript with all author disclosures.

Sandro Beros   Author Signature: S. Beros   Date: 06.03.2026
