# Supplementary material for: Survival in the Third Decade of Uncemented Total Hip Arthroplasty With Small-Diameter Metal-on-Metal Versus Ceramic-on-Conventional Polyethylene Bearings
Source: Arthroplast Today. 2026 Jul 8;40:102078. doi: 10.1016/j.artd.2026.102078 (PMC13380179; doi:10.1016/j.artd.2026.102078)
Supplement: Conflict of Interest Statement for Pourzal [file mmc6.pdf]

CONFLICT OF INTEREST STATEMENT

American Association of Hip and Knee Surgeons

(Adopted from the American Academy of Orthopaedic Surgeons disclosure statement)

The following form **must be filled out completely and submitted by each author (example, 6 authors, 6 forms). All items require a response. If there is no relevant disclosure for a given item, enter "*None.*"**

20 TO 24 YEARS SURVIVAL AFTER UNCEMENTED TOTAL HIP ARTHROPLASTY WITH SMALL-DIAMETER METAL-ON-METAL OR WITH CERAMIC-ON-CONVENTIONAL POLYETHYLENE BEARINGS

1. Royalties from a company or supplier (The following conflicts were disclosed)

2. Speakers bureau/paid presentations for a company or supplier (The following conflicts were disclosed)

   CeramTec at the Annual Meeting of ISTA, 2025

3A. Paid employee for a company or supplier (The following conflicts were disclosed)

3B. Paid consultant for a company or supplier (The following conflicts were disclosed)

3C. Unpaid consultants for a company or supplier (The following conflicts were disclosed)

4. Stock or stock options in a company or supplier (The following conflicts were disclosed)

5. Research support from a company or supplier as a Principal Investigator (The following conflicts were disclosed)

   Zimmer Biomet, Enovis

6. Other financial or material support from a company or supplier (The following conflicts were disclosed)

7. Royalties, financial or material support from publishers (The following conflicts were disclosed)

8. Medical/Orthopaedic publications editorial/governing board (The following conflicts were disclosed)

9. Board member/committee appointments for a society (The following conflicts were disclosed)

Each author must sign AND print or type his/her name, date and submit a separate form

In addition, one BLINDED Conflict of Interest form (no author names used) should be submitted per manuscript with all author disclosures.

Robin Pourzal — 3/11/2026

Author Name (Print or Type) | Author Signature | Date
